# Machine learning to reveal an astute risk predictive framework for Gynecologic Cancer and its impact on women psychology: Bangladeshi perspective

**DOI:** 10.1186/s12859-021-04131-6

**Published:** 2021-04-24

**Authors:** Sayed Asaduzzaman, Md. Raihan Ahmed, Hasin Rehana, Setu Chakraborty, Md. Shariful Islam, Touhid Bhuiyan

**Affiliations:** 1 Department of Computer Science and Engineering, Rangamati Science and Technology University, Vedvedi, Rangamati, Bangladesh; 2 Department of Software Engineering, Daffodil International University, Dhanmondi, Dhaka, Bangladesh; 3 Department of Computer Science and Engineering, Daffodil International University, Dhanmondi, Dhaka, Bangladesh; 4 Department of Information and Communication Technology, Mawlana Bhashani Science and Technology University, Tangail, 1902 Bangladesh; 5 Department of Computer Science and Engineering, Rajshahi University Engineering and Technology, Rajshahi, Bangladesh; 6 Department of Biotechnology and Genetic Engineering, Mawlana Bhashani Science and Technology University, Tangail, Bangladesh

**Keywords:** Gynecological cancer, Significant risk factors, Smart prediction tool, Women psychology, Machine learning, Data mining

## Abstract

**Background:**

In this research, an astute system has been developed by using machine learning and data mining approach to predict the risk level of cervical and ovarian cancer in association to stress.

**Results:**

For functioning factors and subfactors, several machine learning models like Logistics Regression, Random Forest, AdaBoost, Naïve Bayes, Neural Network, kNN, CN2 rule Inducer, Decision Tree, Quadratic Classifier were compared with standard metrics e.g., F1, AUC, CA. For certainty info gain, gain ratio, gini index were revealed for both cervical and ovarian cancer. Attributes were ranked using different feature selection evaluators. Then the most significant analysis was made with the significant factors. Factors like children, age of first intercourse, age of husband, Pap test, age are the most significant factors of cervical cancer. On the other hand, genital area infection, pregnancy problems, use of drugs, abortion, and the number of children are important factors of ovarian cancer.

**Conclusion:**

Resulting factors were merged, categorized, weighted according to their significance level. The categorized factors were indexed using ranker algorithm which provides them a weightage value. An algorithm has been formulated afterward which can be used to predict the risk level of cervical and ovarian cancer in relation to women's mental health. The research will have a great impact on the low incoming country like Bangladesh as most women in low incoming nations were unaware of it. As these two can be described as the most sensitive cancers to women, the development of the application from algorithm will also help to reduce women’s mental stress. More data and parameters will be added in future for research in this perspective.

**Supplementary Information:**

The online version contains supplementary material available at 10.1186/s12859-021-04131-6.

## Highlights of the research

- The Research done on the Dataset of most common Gynecological cancer.
- Analysis of data done by Machine Learning approach.
- Outcome of the research indicates the significant factors and significance level.
- An algorithm has been designed based on the results of the analysis and weightage.
- Algorithm based App outcomes the risk prediction level for gynecological cancer.

## Introduction

Neurodegenerative Disease and post awful neurodegenerative issue are considered as a genuine factor for some major illnesses. It is seen that individuals suffering from neurodegenerative disease disorders have a 55% chance to be affected with cervical cancer [1, 2]. Women who experienced at least 6 side effects of post-traumatic neurodegenerative disease disorder had a more crucial danger of being affected with ovarian cancer [3]. According to WHO, the second leading disease is cancer, it causes 9.6 million death in 2018 [4]. An uncontrolled increase of irregular cells exceeding their regular territory with the ability to attack or potentially spread to different organs is cancer. Among different types of cancer cervical and ovarian cancer is the most prominent hazard to female’s wellbeing [5]. Due to cervical and ovarian cancer every year, over and above 3,00,000 women dies further half a million were diagnosed. Around 5,00,000 women were affected with cervical cancer every year and 274 000 die due to cervical cancer [6].

An assessment [7] expected to perceive risk factors for Lower Limb Lymphedema (LLL) which is a persistent and weakening condition troubling patients who go through lymphadenectomy for gynecologic cancer and to develop a foreseeing model for its happening. To encourage future researches in the field of gynecologic cancer, a model was introduced in a study [8] to predict the risk for psychologic and conduct morbidity. Neurodegenerative disease is linked with diverse neurodegenerative issue, specifically, the pathophysiological importance of pressure in Alzheimer's infection and several diseases. Some previous studies also showed that neurodegenerative disease spurs on cervical and ovarian cancer [9, 10]. National Cancer Institute summarizes that cervical tumor constitutes in the cervix, a body part associating with uterus and vagina [11, 12] Human Papillomavirus (HPV) is the main reason behind cervical cancer [13] For the well-known impact of HPV on cervical cancer, an examination [14] was made which audits the articles based on the information on HPV and cervical cancer among Malaysian inhabitants before and after the usage of HPV antibody programs. In [15] a research has been conveyed a study where he found cervical slowly develops without showing any indication at the beginning seemingly hard to discover but can be noticed with respective pap tests. A study was made with 70 patients in china with insomnia provoked by cervical cancer [16]. This irregularly checked preliminarily selected patients with sleep deprivation that arises or exacerbated by cervical disease [17]. A survey was carried out in [16] using the data of Nurses' Health Study found a substantial relationship between treatment for PTSD (Post Traumatic Stress Disorder) and the growth of ovarian cancer. According to American Cancer Institute, ovarian cancer is supposed to start in the ovaries but recent knowledge exhibits that numerous ovarian tumors may begin in the fallopian tubes, which holds two ovaries after the body of the uterus. Likely cervical, ovarian cancer is hard to recognize [18]. The ovaries lie profound inside the abdominopelvic pit, making them hard to view or feel [19, 20]. The epithelial ovarian disease stays an exceptionally dangerous (Hunn & Rodriguez, 2012). A feasible study [20] offers risk management options of screening and prevention of ovarian cancer. It has been proved, due to the amendment of the p53 gene, cells affected due to neurodegenerative diseases persuades ovarian epithelial cancer [21]. A study [22] presents data which shows, emission of incendiary proteins in ovarian cancer cell were prompted by stress hormones.

According to [23], Cancer is the main source of death. Recent studies [15] suggested that the event of lung cancer has expanded quickly and turned the most widely recognized disease worldwide. A full research was made in [24] to build up a framework that can be utilized by an individual to test his risk level of Lung Cancer. And utilizing the acquired knowledge an experiment was able to predict the risk level of lung cancer [25]. Another research was carried out to build up a system that can be utilized by an individual for knowing his risk level of skin cancer [26]. Presently Type-1 Diabetes is also a shocking sickness in Bangladesh. Type 1 diabetes, which is known as adolescent diabetes or insulin-subordinate diabetes, is an interminable condition where the pancreas delivers mostly zero insulin. Information has been gathered from Dhaka dependent on a particular questionnaire to show the association and criticalness among the degree of elements [27].

In this paper “Introduction” section, represents the risk prediction models and their techniques behind prediction were discussed elaborately. We conduct experiments on three datasets in “Material and methods” section and it was conducted with the help of knowledge discovery. Their efficiency in prediction is shown with a set of figures and tables. This section also contains the output of our research which is the mobile application that we have prepared for risk prediction. For preparing this application at first, we’ve made an equation to differentiate the risk level and prepared an algorithm. The algorithm is provided in “Material and methods” section. Finally, this work is concluded in “Results and discussion” section and future work is proposed afterward.

## Material and methods

This paper uses popular data mining and machine learning models were compared with metrics such as accuracy, precision, recall, F1, support [28] This was found using a sklearn library [29] of python and orange machine learning and data mining toolkit. We’ve further proposed an equation based on the difference between the result-metrics of these two toolkits. Using apriori algorithm correlation among the significant factors which describe the dependency among the factors were described. Ranker algorithm is the most efficient algorithm can be used to rank the features for indexing than BestFirst or, GreedyStepwise. Feature selection was performed using the ranker algorithm. Key factors on the data analysis were derived for all the evaluators of the ranker algorithm [30]. Afterward, it was compared among them and the worthiest attribute was obtained. For prediction, it is important to find out the significant factors. Here, the importance of factors has been gathered according to info gain, gain ratio, gini index [31, 32].

### Data collection

In total 866 data were collected from various diagnosis centers of patients suffering from diseases like ovarian, cervical, and stress (Mental) disorder. Data of 161 female patients were collected from those who were experiencing cervical cancer. Data were collected from a set of questionnaires which includes 25 attributes. 522 patients of ovarian cancer were interviewed with set questions which contain 46 risk factors. The set of Questioner for Cervical Cancer and Ovarian Cancer has been provided as (Additional File 1 and Additional File 2) Respectively.

### Data preprocessing

Data cleaning, data integration, data selection, data transformation are four leading tasks of data pre-processing to convert the dataset from noisy, inconsistent data to a format suitable for mining and learning predictability. The corrupted or distorted data with meaningless information those are provided by the patients while answering the questionnaire are noisy data. In the data cleaning phase those noisy, conflicting and inconsistent data were removed. Valuable data were joined in the data integration phase. Data suited for the analysis were retrieved from the dataset in the data selection phase. Finally, in data transformation, data were converted to proper structures which fits for data mining and machine learning analysis. To ignore the collision of the data a small amount of data was altered.

### Evaluation of the performance of machine learning models

In this lesson, eight classifiers are known as SVM, Random Forest, Logistic Regression, AdaBoost, Naïve Bayes, Neural Network, kNN, CN2 rule Inducer were used for evaluation with orange and almost 10 classifiers namely SVM, Random Forest, Logistic Regression, AdaBoost, Naïve Bayes, Neural Network, kNN, Gaussian Process, Decision Tree, Quadratic Classifier were used for assessment of machine learning models. In this context, the performance was measured using standard metrics like the area under the ROC curve (AUC), precision, classification accuracy, recall, specificity, F measure, support. A decision tree was constructed with the important factors of cervical, ovarian, and stress datasets.

Performance measures: Classification accuracy rates for the datasets were analyzed. For each dataset, two classes were identified namely positive and negative. There are four possibilities for a single prediction e.g., true positive, true negative, false positive, false negative. True positive and true negatives are described as how many correct predictions were made. False-positive and false-negative provides how many incorrect predictions were made of positive and negative classes when they belong to positive and negative classes.

Accuracy: It defines the number of correct predictions that were correctly classified from the total number of predictions in ratio Eq. (1)

1 $$accuracy = \frac{TP + TN}{{TP + TN + FP + FN}}$$

Precision: Explains the number of positive predictions that were correctly classified by the classifier from the total number of positive predictions Eq. (2)

2 $$precision = \frac{TP}{{TP + FP}}$$

Recall: Characterizes the fraction of correct positive predictions of the whole equation Eq. (3)

3 $$recall = \frac{TP}{{TP + FN}}$$

F-measure: It predicts the average value of precision and recalls Eq. (4)

4 $$F - measure = \frac{{2 \times \left( {precision \times recall} \right)}}{precision + recall}$$

Specificity: Measures the number of whole negative prediction those are correctly identified by the classifier Eq. (5)

5 $$Specificity = \frac{TN}{{TN + FP}}$$

Support: Defines the number of datasets that were analyzed after training and splitting the whole dataset. From my analysis, it is seen that sklearn uses only 24% of the whole dataset that was used Eq. (6)

6 $$Support = 0.24 \times no.\,of\,data \in the\,dataset$$

The first and foremost concept to judge the probability is to find significant factors through various analyses. The study was undertaken with lots of derivatives and algorithms to find out the significant factors. The level of important factors was acquired using information gain, gain ratio, gini index. Information Gain: It is a measurement of the decrease in uncertainty. It is estimated from entropy. Entropy is the measurement of the probability of changeability of the processed information. The higher the entropy, the harder it is to make any determinations from that data Eq. (7)

7 $$\begin{aligned} entropy\left( {p_{1} ,p_{2} \ldots p_{n} } \right) & = - p_{1} \log p_{1} - p_{2} \log p_{2} \ldots \ldots - p_{n} \log p_{n} \\ E\left( x \right) & = \mathop \sum \limits_{i = 1}^{c} - p_{i} \log p_{i} \\ E\left( {T,X} \right) & = \mathop \sum \limits_{c \in X} P\left( c \right)E\left( c \right) \\ \end{aligned}$$

Summation of the feature probability of values times the log probability of the same label. By deducting the value of label and features from the entropy of label information gain is obtained Eq. (8)

8 $$Gain\left( {T,X} \right) = E\left( T \right) - E\left( {T,X} \right)$$

Gain Ratio: Modifies information gain by taking ignored information into account including the number and sizes of the branches that reduces the bias of information gain Eqs. (9, 10)

9 $$SplitEntropy\left( {T,X} \right) = \mathop \sum \limits_{c \in X} - \frac{{T_{x} }}{T}\log \frac{{T_{x} }}{T}$$

10 $$GainRatio = \frac{{Gain\left( {S,A} \right)}}{{SplitEntropy\left( {S,A} \right)}}$$

Gini Index: It measures the impurity of a single feature. It is obtained by subtracting the sum of squared probabilities from one Eqs. (11, 12)

11 $$Gini\left( X \right) = 1 - \mathop \sum \limits_{i = 1}^{c} \left( {p_{i} } \right)^{2}$$

12 $$\Pr ediction\, difference = (\sum highest - \sum lowest) \div 4$$

From information gain, we acquired the certainty of individual features for a specific label. Gain ratio provides us the same including the intrinsic information of the dataset. Gini index provides how much filthy an individual factor is. All of these values are gathered in terms of 0 and 1. Equation analyzing the chi-square test and results of feature selection evaluators we found out the most significant factors which are working behind cervical and ovarian cancer in connection with stress. Then these factors were given different scores based on their significance level. Afterward, the Eq. 12. was defined to separate the risk levels of an individual.

## Results and discussion

The results and discussion section has been discussed and analyzed in this section. Some data mining and machine learning techniques have been applied. We have analyzed the datasets of cervical and ovarian and found common pattern significant attributes. The attributes were selected as common and highly significant factors and correlated with the possibilities of Cervical or Ovarian cancer. Table 1 shows the values of Info Gain, Gini Index, and the Gain ratio of the parameters. Table 1 also shows the Chi-Square test values along with ranking values. Chi-square values were acquired from statistical analysis and ranked values are found from the Data Mining algorithm. A different analysis of the parameters was conducted with different attribute evaluator and has been shown in Table 2.

**Table 1:** Attribute values for info gain, gain ratio, Gini index, Chi square and ranker value

Attributes	Info. gain	Gain ratio	Gini index
Problem during pregnancy	0.408	0.414	0.25
Abortion	0.355	0.355	0.225
Have infection in the genital area	0.342	0.356	0.213
Affected by breast cancer	0.251	0.278	0.157
Estrogen pill taken after menopause	0.237	0.291	0.142
Ever had a hysterectomy	0.228	0.268	0.141
Condom/diaphrame	0.296	0.385	0.182
Any birth control pill	0.221	0.232	0.143
Use napkin	0.22	0.263	0.135
Pregnency after 35	0.212	0.245	0.133
Pap test	0.211	0.246	0.132
Children after 35	0.208	0.237	0.131
Takes hormone after menopause	0.206	0.251	0.127
Take adequate fruit	0.185	0.187	0.122
Age	0.545	0.312	0.302
Oral contraception	0.253	0.316	0.158
Education	0.192	0.102	0.113
Age of husband	0.579	0.372	0.314
Cancer vaccine taken	0.007	0.031	0.005
First sex age	0.453	0.453	0.219
Marital status	0.041	0.099	0.017
No of children	0.634	0.481	0.321
No of sex partner	0.065	0.115	0.029 + A1:D24

**Table 2:** Attribute values for different algorithm ranking with various sub-evaluator

SerialRank	Parameters	Rel Att Eval	Sym Un Att	Info Gain Att	OneR Att
			Eval	Eval	Eval
1	Problem during pregnancy	1	1	1	1
2	Abortion	NA	3	3	3
3	Have infection in the genital area	2	2	2	2
4	Affected by breast cancer	4	4	4	4
5	Estrogen pill taken after menopause	15	5	5	9
6	Ever had a hysterectomy	12	6	6	15
7	Condom or diaphragm	7	7	7	6
8	Any birth control pill	5	10	9	5
9	Use napkin	9	11	11	12
10	Pregnancy after 35	14	12	14	10
11	Pap test	18	15	15	7
12	Children after 35	10	16	17	14
13	Takes hormone after menopause	19	17	18	18
14	Take adequate fruit	17	19	19	17
15	Age	12	18	20	19
16	Oral contraception	11	20	21	21
17	Education	NA	21	22	20
18	Age of husband	NA	22	24	22
19	Cancer vaccine taken	NA	24	NA	24
20	First sex age	21	NA	NA	NA
21	Marital status	NA	NA	NA	NA
22	Number of children	20	NA	NA	NA
23	Number of sex partner	22	NA	NA	NA
24	Long term pressure	24	NA	NA	NA

The serial position of the attributes in different attributes refers to the position of the attribute in the ranking table of corresponding sub evaluators.

**Fig. 1:**
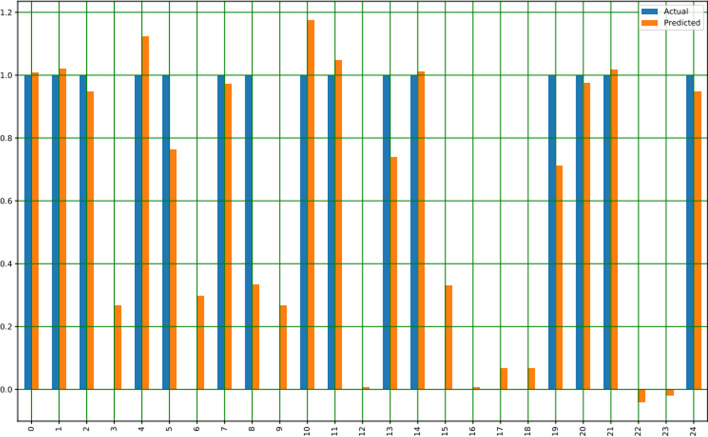
Linear regression probabilities for different parameters

**Fig. 2:**
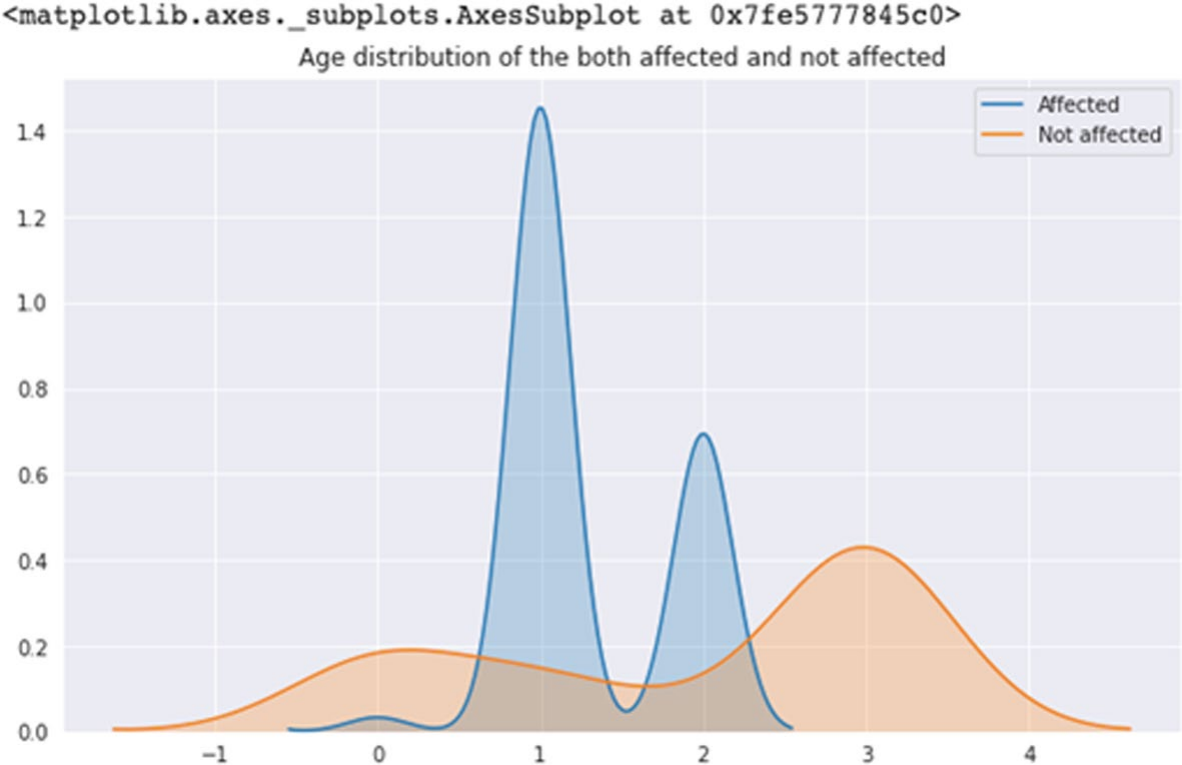
KDE Plot for both affected and not affected people according to age distribution

**Fig. 3:**
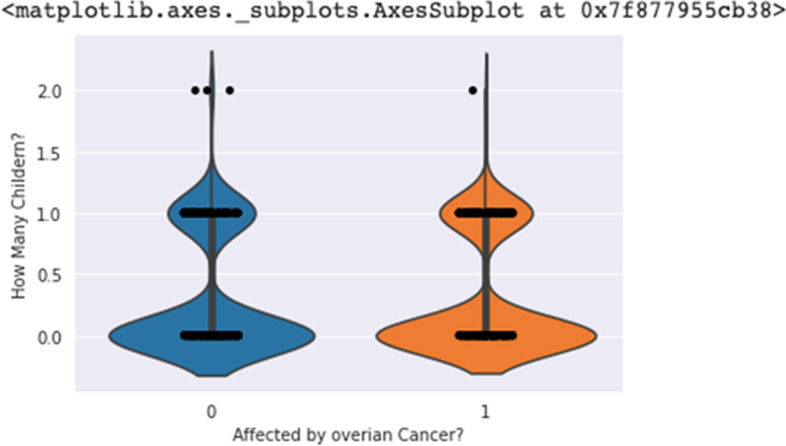
Violin plot for ovarian cancer versus no of children

Figure 1 indicated the logistic regression analysis values of the actual and predicted data. In x-axis total of 25 values represents 25 separate parameters and the serial of the parameters are as same as the serial of Table 2. The 24th parameter is related to the mental health or stress of women. The following figure depicts that the higher no of affected women has been shown by linear logistic regression. KDE plot of Fig. 2 tends to estimate the probability distribution functions of affected and non-affected women. The sub-parameters were assigned numeric values e.g., 1 represents above 60, 2 represents 46–60 etc. The affected plot varies from 1 to 1.45 means a higher number of affected women rely on age more than 60 and similarly, 2–0.7 points describe that the second most affected women had an age of 46–60. Violin plot visualizes the distribution of data and its probability density as shown in Fig. 3. Children of 3–5 or above 5 suffer from cervical or ovarian cancer according to the violin plot of Fig. 3. Violin of 0 level means having a higher number of children which had those cancers.

Tables 3, 4 and 5 shows the accuracy of the data for Mental Stress, Ovarian cancer and Cervical cancer according to different machine learning classifiers and also displays the classification accuracy, F1, precision metrics which can be used to compare the machine leaning models. The accuracy level is organized between 0 and 1. The prediction accuracy of the model increases with the value getting closer to 1. It also indicates the significance. All the significant factors and sub-factors of the diseases were first indexed with the help of ranker algorithm and then combined to get a whole picture which is later used for anticipation. The compared notable features with weightage values have been displayed in Table 6. Finally, an algorithm has been developed based on the weightage values of Table 6.

**Table 3:** Accuracy of stress according to different machine learning algorithms

Model	Tool	AUC	CA	F1	Precision	Recall	Specificity	Support
SVM	Orange	0.746	0.744	0.698	0.736	0.744	0.462	–
	Sklearn	0.615		0.6	0.59	0.62	–	39
Random	Orange	0.746	0.725	0.707	0.705	0.725	0.525	–
Forest	Sklearn	0.692		0.7	0.73	0.69	–	39
Logistic	Orange	0.796	0.769	0.752	0.757	0.769	0.579	–
Regression	Sklearn	0.769		0.77	0.76	0.77	–	39
AdaBoost	Orange	0.744	0.788	0.785	0.783	0.788	0.683	–
	Sklearn	0.79		0.79	0.79	0.79	–	39
Naïve	Orange	0.753	0.763	0.763	0.763	0.763	0.672	–
Bayes	Sklearn	0.785		0.78	0.79	0.78	–	39
Neural	Orange	0.767	0.75	0.747	0.745	0.75	0.631	–
Network	Sklearn	0.692		0.57	0.48	0.69	–	39
kNN	Orange	0.646	0.669	0.627	0.618	0.669	0.394	–
	Sklearn	0.615		0.6	0.59	0.62	–	39
CN2 rule	Orange	0.721	0.694	0.696	0.699	0.694	0.595	–
Inducer	Sklearn		–		–		–	–
Decision	Orange	0.7	0.713	0.717	0.723	0.713	0.639	–
Tree	Sklearn	0.692		0.7	0.71	0.69	–	39
Quadratic	Orange		–		–		–	–
Classifier	Sklearn	0.744		0.7	0.75	0.74	–	39

**Table 4:** Accuracy of ovarian cancer according to different machine learning algorithms

Model	Tool	AUC	CA	F1	Precision	Recall	Specificity
SVM	Orange	0.883	0.742	0.704	0.741	0.742	0.8
	Sklearn	0.861	–	0.85	0.87	0.85	–
Random	Orange	0.868	0.755	0.745	0.744	0.755	0.841
Forest	Sklearn	0.972	–	0.97	0.97	0.98	–
Logistic	Orange	0.863	0.735	0.721	0.72	0.735	0.828
Regression	Sklearn	1	–	1	1	1	–
AdaBoost	Orange	0.86	0.742	0.737	0.737	0.742	0.828
	Sklearn	1	–	1	1	1	–
Naïve	Orange	0.851	0.621	0.627	0.642	0.621	0.836
Bayes	Sklearn	0.958	–	0.96	0.96	0.96	–
Neural	Orange	0.847	0.718	0.719	0.721	0.718	0.838
Network	Sklearn	0.986	–	0.99	0.99	0.98	–
kNN	Orange	0.845	0.735	0.725	0.723	0.735	0.833
	Sklearn	0.861	–	0.85	0.87	0.85	–
CN2 rule	Orange	0.821	0.674	0.675	0.676	0.674	0.815
Inducer	Sklearn	–	–	–	–	–	–
Decision	Orange	–	–	–	–	–	–
Tree	Sklearn	0.986	–	0.99	0.98	0.99	–
Quadratic	Orange	–	–	–	–	–	–
Classifier	Sklearn	0.431	–	0.3	0.22	0.5	–

**Table 5:** Accuracy of cervical cancer according to different machine learning algorithms

Model	Tool	AUC	CA	F1	Precision	Recall	Specificity	Support
SVM	Orange	0.883	0.742	0.704	0.741	0.742	0.8	–
	Sklearn	0.861	–	0.85	0.87	0.85	–	72
Random forest	Orange	0.868	0.755	0.745	0.744	0.755	0.841	–
	Sklearn	0.972	–	0.97	0.97	0.98	–	72
Logistic regression	Orange	0.863	0.735	0.721	0.72	0.735	0.828	–
	Sklearn	1	–	1	1	1	–	72
AdaBoost	Orange	0.86	0.742	0.737	0.737	0.742	0.828	–
	Sklearn	1	–	1	1	1	–	72
Naïve Bayes	Orange	0.851	0.621	0.627	0.642	0.621	0.836	–
	Sklearn	0.958	–	0.96	0.96	0.96	–	72
Neural network	Orange	0.847	0.718	0.719	0.721	0.718	0.838	–
	Sklearn	0.986	–	0.99	0.99	0.98	–	72
kNN	Orange	0.845	0.735	0.725	0.723	0.735	0.833	–
	Sklearn	0.861	–	0.85	0.87	0.85	–	72
CN2 rule inducer	Orange	0.821	0.674	0.675	0.676	0.674	0.815	–
	Sklearn	–	–	–	–	–	–	72
Decision tree	Orange	–	–	–	–	–	–	–
	Sklearn	0.986	–	0.99	0.98	0.99	–	72
Quadratic classifier	Orange	–	–	–	–	–	–	–
	Sklearn	0.431	–	0.3	0.22	0.5	–	72

**Table 6:** Weightage value of parameters

Parameters	Sub-parameters	Weight/score
Problem during pregnancy	Yes	14
	No	13
Abortion	Yes	13
	No	12
	No	11.5
Affected by breast cancer	Yes	11.5
	No	10.5
Estrogen pill taken after menopause	No	10
	Yes	10.5
Ever had a hysterectomy	No	10
	Yes	9.5
Condom or diaphragm	No	9.5
	Yes	9
Any birth control pill	No	8.5
	Yes	9
Use napkin	No	8.5
	Yes	8
Pregnancy after 35	Yes	8
	No	7.5
Pap test	No	7.5
	Yes	7
Children after 35	Yes	7
	No	6.5
Takes hormone after menopause	No	6
	Yes	6.5
Take adequate fruit	No	6
	Yes	5.5
	Above 60	5.5
Age	46–60	5.5
	30–45	5.25
	Below 30	5
Oral contraception	Yes	5
	No	4.5
	Undergraduate	4
Education	Primary	4.25
	Secondary	4.25
	Illiterate	4.5
	Above 60	4
Age of husband	40–60	3.65
	Below 30	3.8
	30–45	3.5

After analyzing the significances of the factor of cervical, ovarian, and stress we have derived an algorithm for predicting the risk levels of the diseases which is shown below,

- Step 1. Start
- Step 2. read weights
- Step 3. total_weights ← $$\sum weights$$
- Step 4. prediction_difference ← 
- Step 5. if total_weights <  = prediction_difference + $\sum lowest$ then print LOW RISK
- Step 6. else if total_weights <  = (prediction_difference*2) + $\sum lowest$ then print MEDIUM RISK
- Step 7. else if total_weights <  = (prediction_difference *3) + $\sum lowest$ then print HIGH RISK
- Step 8. else print VERY HIGH RISK
- Step 9. Stop

**Fig. 4:**
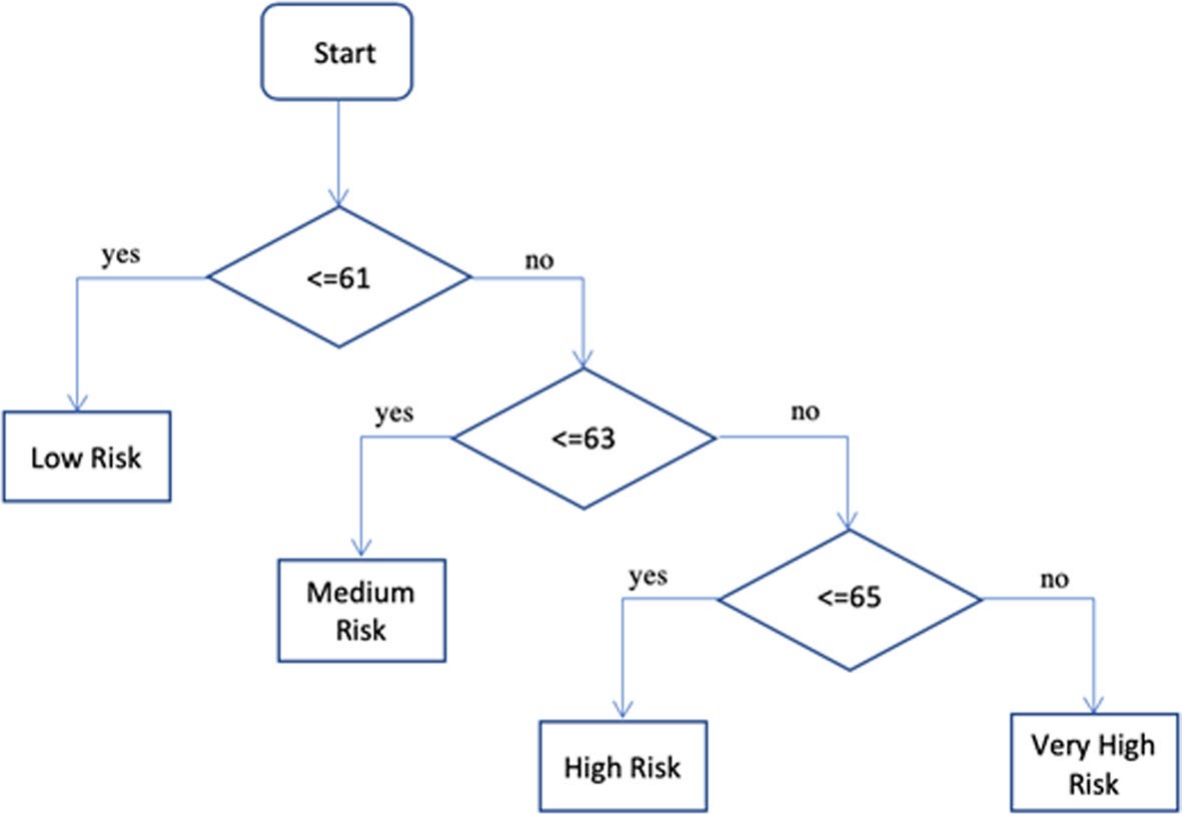
Flowchart of designed algorithm

**Fig. 5:**
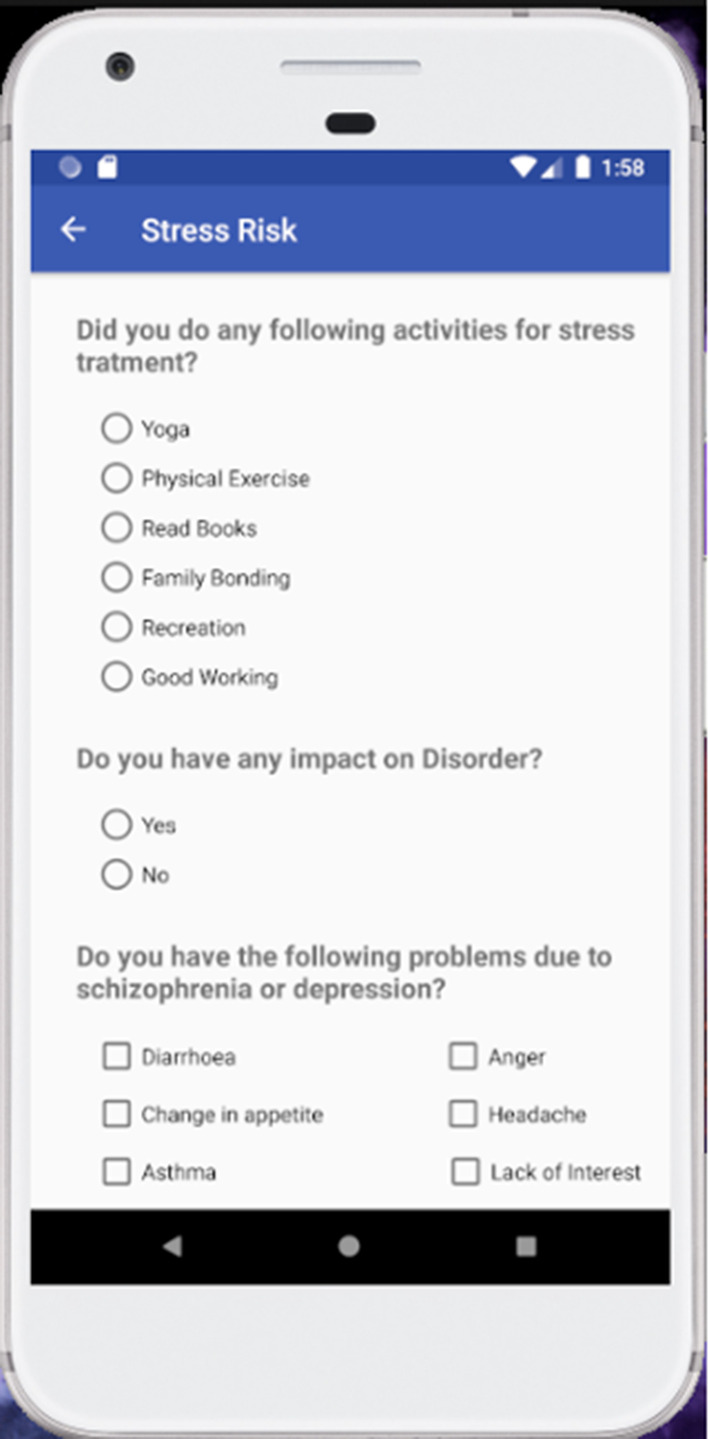
Android application for cancer risk prediction (women health)

**Fig. 6:**
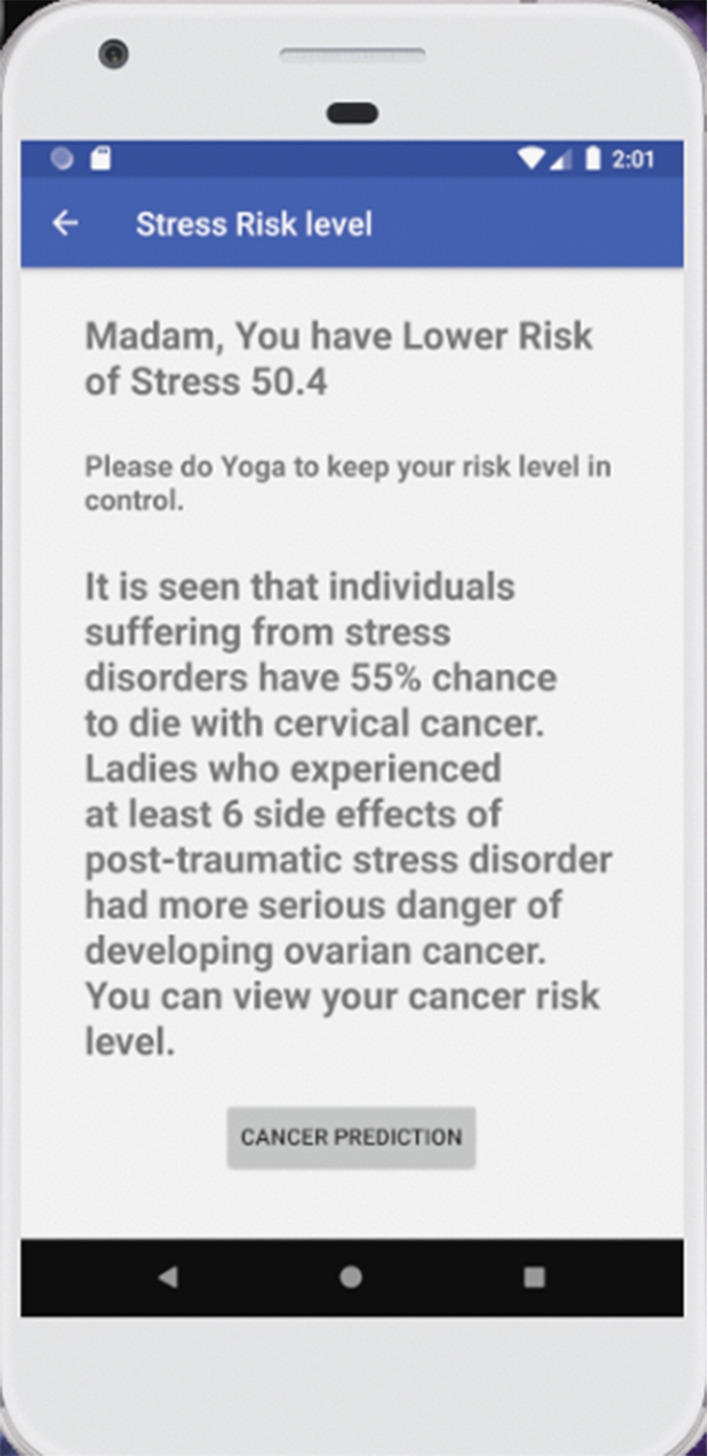
Android application results for cancer risk prediction (women health)

The flowchart of the algorithm has been shown in Fig. 4. With the help of the above algorithm, we found out the respective flowcharts for the diseases. At last, we put all the flowcharts and significant factors together to elicit the superior significant factors. Afterward combining cervical, ovarian, and stress factors we have drawn the flowchart for all of them. From those flowcharts and using the algorithm that predicts from the significant factors we have prepared an application shown in Figs. 5 and 6. The application is well prepared with the intention to store the future data in the cloud provided by the users and use that information for further investigation. We got better results with the sample data after training and testing of the models which makes us confident to use the for predicting those diseases. After utilizing the upcoming data, we will be able predict the diseases much more accurately.

The combined decision trees of cervical and ovarian cancer were pointed in Fig. 4, which indicates there are maximum chances to be infected by cervical virus if a woman had more than 2 children. If she had 1–2 children and her first intercourse was made when she was less than 16 years old than there are also maximum possibility of cervical cancer. In here, a decision is made taking 6 precious factors for emerging cervical cancer. Likewise, taking 15 parameters a decision is made to find out the risk of appearing ovarian cancer. The most risk factors are abortion, age of husband, alcohol consumption, etc.

## Conclusion

Cervical and ovarian cancers are the dominant causes of women’s demise in Bangladesh. The majority of the people are unconscious of it. Death is inescapable because of cervical and ovarian cancer. From the findings, we got the evidence that immune response may be damaged due to neurodegenerative disease may even enhance the development of cancer. In this scrutiny, risk factors of cervical and ovarian cancer were analyzed carefully. Here, data mining and machine learning models like SVM, Random Forest, Logistics Regression, AdaBoost, Naïve Bayes, Neural Network, kNN, CN2 rule, Decision Tree, Quadratic Classifier have been used and those models were compared with two different tools. The obtained result for neurodegenerative disease shows that AdaBoost performed the best with a classification accuracy of 78.8% in orange and 79% in Sklearn. In the case of cervical cancer, Logistics Regression provides the best score of 84.8% and with Sklearn we’ve got 79.3%. On the other hand, SVM shows the best accuracy of 88.3% in orange, and the decision tree provides 98.6% classification accuracy in Sklearn for ovarian cancer. Based on all the analyses finally, an algorithm along with a smart app was developed by the weightage values generated from the analysis. Future works can be done by improving the dataset size, tuning parameters, and more effective analysis.

## Summary of the work

Cervical and ovarian cancer is the one of the most frightening disease among females in the low approaching nation like Bangladesh. The community of Bangladesh are lacking behind in education and awareness about these two cancers. Previous studies have found that stress is somehow influencing these two cancers. Any kind of prediction of cervical and ovarian cancer among Bangladeshi female’s is not available in this modern age. Purpose: To find out the association between factors and the most significant factors of stress, cervical, ovarian cancer. Contribute a prediction on befalling cervical and ovarian cancer based on their worthy factors as well as stress parameters. Methods: A case control study has been made on 298 patients of cervical and 522 patients of ovarian cancer. Cases of 197 and control of 100 were considered for cervical cancer. In case of ovarian cases of 267 and control of 254 beheld for data mining analysis. For analyzing performance of several machine learning models e.g. Logistics Regression, Random Forest, AdaBoost, Naïve Bayes, Neural Network, kNN, CN2 rule Inducer, Decision Tree, Quadratic Classifier were compared with their standard metrics. For certainty info gain, gain ratio, gini index were revealed of both cervical and ovarian cancer. Attributes were ranked using different feature selection evaluators. Then the most significant analysis was made with the significant factors. Factors like children, age of first intercourse, age of husband, pap test, age are significantly higher factors of cervical cancer. On the other hand, genital area infection, pregnancy problem, use of drugs, abortion, number of children important factors of ovarian cancer. The analysis was made with significant factors of stress, cervical and ovarian cancer that will help us to predict the risk of occurring cervical or ovarian cancer and may help to abate the cancer not only from Bangladesh but all over the world. After analyzing a weightage table has been created to make an algorithm which can predict risk level of two fatal cancer of Women (cervical and Overian) along with mental health.

## Methods used in manuscripts

All methods were carried out in accordance with relevant guidelines and regulations.

## Informed consent/waiver on informed consent

The need for written informed consent was waived by the Institutional Review Board/ethics committee of Department of Software Engineering, Daffodil International University. Because the Concern Hospitals dataset consisted of de-identified secondary data for research purposes. Verbal Consent from all the participants has been taken that no information will be disclosed and the dataset will only be used for research purpose.

## Supplementary Information

**Additional file 1.** Sample Questioner for Cervical Cancer Data Collection.

**Additional file 2.** Sample Questioner for Ovarian Cancer Data Collection.

## Data Availability

Data will be available by the corresponding author on request for research purpose only.
