# Supplementary material for: Machine learning to reveal an astute risk predictive framework for Gynecologic Cancer and its impact on women psychology: Bangladeshi perspective
Source: BMC Bioinformatics. 2021 Apr 24;22:213. doi: 10.1186/s12859-021-04131-6 (PMC8066470; doi:10.1186/s12859-021-04131-6)
Supplement: Supplementary file 1 — Additional file 1. Sample Questioner for Cervical Cancer Data Collection. [file 12859_2021_4131_MOESM1_ESM.docx]

**QUESTIONEER FOR CERVICAL CANCER**

**Dept. of Information and Communication Technology and Dept of Biotechnology and Genetic Engineering**

**Mawlana Bhashani Science and Technology University**

**Santosh, Tangail-1902**

**A Survey on Risk Factors of Cervical Cancer in Bangladesh**

The questionnaire will be applied only for academic purpose. The respondents can answer the questions without any hesitation

1. Name: (Full Name) ……………………………Sumi Akter………………………………..

2. Area of Residence: a. Rural b. Urban c. Suburban d. Other

3. Age: a. below 30 b. 31-45 c. 46-60 d. Above 60

4.Marital status: a. married b. unmarried c. divorce d. Separate e.Widow

5.Age of Husband: a.bellow 30 b.31-45 c.45-60 d.Above 60

6. Education Level: a. Illiterate b. Primary c. Secondary d. Undergraduate or above

7. Occupation: a. Business b. Govt. service c. Farmer d .Unemployed

e. Housewife f. Private jobs g. Shop Keeper

8. Family Members: a. 1-3 b. 3-5 c. 6 and above

9. Social class/ status: a. Rich b. Upper middle c. Lower middle d. Poor e. Destitute

10.Family Income (Monthly): a. 3000> less b. 4000-8000 c. 9000-18000 d. 19000-30000e. 31000<above

11. Expenses (Monthly): a. Below average b. Reasonable c. Extensive/ High

12.Do you have any knowledge of cervical cancer: a.yes b.no

13.Have you ever had any type of cancer : a. yes b. no

14. If had please specify: a.blood b.lung c.brain d.liver e.other

15.Have you ever taken a vaccine for cervical cancer: a.yes b.no

16. Have you ever had a hysterectomy (surgical removal of uterus): a. yes b. no

17.Do you eat adequate amounts of fruits and vegetables: a.yes b.no

18. Do you smoke : a. yes b. no

19.How many male sexual partner have you had in your life time : a. 0 b. 1-2 c.3+

20.How old were you when you first had a sex with a male partner: a. below 16 b. above 16

21. Throughtout your life, have you consistently used a condom or diaphragm during sex : a. yes b.no

22.Do you use oral contracepties: a.yes b.no

23. How many children you have given birth: a. 1-2 b. 3-5 c. above 5

24. Have you ever had an STI (Sexually transmitted infections) : a. yes b.no

If yes then what types ? a. HPV b. Herpes c. Gonorrhea d. HIV/AIDS e.Chlamydia

25. Have you had a pap test within the last three years : a. yes b.no

26.Have you or your mother were given diethylstilbestrol: a,yes b.no

27.Has anyone in your immediate family had a cervical cancer: a.yes b.no

If had please specify: a.mother b.sister c.aunty d.grandmother

28. Are you affected by cervical cancer: a.yes b.no

The Answers were given by green mark. Data was collected from different age of Women who are affected/not affected by Cervical Cancer. Data is Kept secret and will only be available for research.
