# Supplementary material for: Machine learning to reveal an astute risk predictive framework for Gynecologic Cancer and its impact on women psychology: Bangladeshi perspective
Source: BMC Bioinformatics. 2021 Apr 24;22:213. doi: 10.1186/s12859-021-04131-6 (PMC8066470; doi:10.1186/s12859-021-04131-6)
Supplement: Supplementary file 2 — Additional file 2. Sample Questioner for Ovarian Cancer Data Collection. [file 12859_2021_4131_MOESM2_ESM.docx]

This information must be added in the chart for survey on ovarian cancer.

1.Name: (Full Name) ……………………………………………………………..

2. Area of Residence: a. Rural b. Urban c. Suburban d. Other

3.Marital status: a. married b. unmarried c. divorce d. Separate e.Widow

4.Age of Husband: a.bellow 30 b.31-45 c.45-60 d.Above 60

5. Education Level: a. Illiterate b. Primary c. Secondary d. Undergraduate or above

6. Occupation: a. Business b. Govt. service c. housewife d .Unemployed

e. Housewife f. Private jobs g. Shop Keeper

7. Family Members: a. 1-3 b. 3-5 c. 6 and above

8. Social class/ status: a. Rich b. Upper middle c. Lower middle d. Poor e. Destitute

9.Family Income (Monthly): a. 3000> less b. 4000-8000 c. 9000-18000 d. 19000-30000e. 31000<above

10. Expenses (Monthly): a. Below average b. Reasonable c. Extensive/ High

11.Have you ever had any type of cancer : a. yes b. no

12. If had please specify: a.blood b.lung c.brain d.liver e.other

14. Have you ever had a hysterectomy (surgical removal of uterus): a. yes b. no

15.How many male sexual partner have you had in your life time : a. 0 b. 1-2 c.3+

16.How old were you when you first had a sex with a male partner: a. below 16 b. above 16

17. Throughtout your life, have you consistently used a condom or diaphragm during sex : a. yes b.no

18.Do you use oral contracepties: a.yes b.no

19. How many children you have given birth: a. 1-2 b. 3-5 c. above 5

20. Have you ever had an STI (Sexually transmitted infections) : a. yes b.no

If yes then what types ? a. HPV b. Herpes c. Gonorrhea d. HIV/AIDS e.Chlamydia

21. Have you had a pap test within the last three years : a. yes b.no

22. Ever had any cancer : a. Yes b. No

23. Knowledge about cancer : a. Yes b. No

24. Marital status : a. Divorced b. Married c. Separate d. Widow

25. education : a. Illiterate b. Primary c. Secondary d. Under graduate/above
